# Guanabenz modulates microglia and macrophages during demyelination

**DOI:** 10.1038/s41598-020-76383-w

**Published:** 2020-11-09

**Authors:** Kaitlyn Koenig Thompson, Stella E. Tsirka

**Affiliations:** Department of Pharmacological Sciences, Stony Brook University, Stony Brook, NY 11794-8651 USA

**Keywords:** Multiple sclerosis, Mechanisms of disease

## Abstract

Multiple sclerosis (MS) is an autoimmune disease characterized by infiltration of peripheral immune cells into the central nervous system, demyelination, and neuronal damage. There is no cure for MS, but available disease-modifying therapies can lessen severity and delay progression. However, current therapies are suboptimal due to adverse effects. Here, we investigate how the FDA-approved antihypertensive drug, guanabenz, which has a favorable safety profile and was recently reported to enhance oligodendrocyte survival, exerts effects on immune cells, specifically microglia and macrophages. We first employed the experimental autoimmune encephalomyelitis (EAE) model and observed pronounced immunomodulation evident by a reduction in pro-inflammatory microglia and macrophages. When guanabenz was administered in the cuprizone model, in which demyelination is less dependent upon immune cells, we did not observe improvements in remyelination, oligodendrocyte numbers, and effects on microglial activation were less dramatic. Thus, guanabenz may be a promising therapeutic to minimize inflammation without exerting severe off-target effects.

## Introduction

Multiple sclerosis (MS) is an autoimmune disease of the central nervous system (CNS), affecting approximately 2.5 million people worldwide^1,2^. Symptoms, such as motor dysfunction and fatigue, arise from the formation of lesions in the brain and/or spinal cord. Lesions are typically multifocal and comprised of inflammation, demyelination, blood–brain barrier breakdown and peripheral immune cell infiltration, loss of oligodendrocytes, and axonal degeneration^2,3^.

There is currently no cure for MS, however, there are several FDA-approved disease-modifying therapies (DMTs) that decrease relapse rate and delay disease progression. These therapies are immunomodulatory in nature and minimize CNS inflammation through various mechanisms ranging from general immunosuppression to targeted prevention of immune cell infiltration into the CNS^4^. Approved DMTs are not without limitations. Adverse effects can severely impact patients’ quality of life or be life-threatening themselves. Further, these therapies have varying efficacies patient-to-patient and none of the approved agents are able to reverse or halt disease progression^5^. Therefore, there remains a critical need to characterize new immunomodulatory drugs with fewer side effects as well as therapies that address repair mechanisms.

A recent study found that guanabenz (2,6-dichlorobenzylidene amino guanidine; Gz), an FDA-approved anti-hypertensive, has the potential to be repurposed as an MS repair drug as it enhances the integrated stress response (ISR), promoting oligodendrocyte survival in experimental autoimmune encephalomyelitis (EAE), an animal model of MS^6^. Some immunomodulatory effects were also observed, with fewer immune cells present in the spinal cord of EAE animals administered Gz daily^6^. There have since been reports that Gz exerts anti-inflammatory activity in other pathological settings such as latent *Toxoplasma gondii* infection^7^, d-Galactosamine/LPS-induced liver damage^8^, and a toxin-induced model of systemic lupus erythematosus (SLE)^9^. In vitro work has demonstrated that Gz directly downregulates pro-inflammatory genes in LPS-stimulated primary macrophages and dendritic cells^8,10^. Taken together, it is possible that Gz exerts anti-inflammatory effects to a greater extent than previously investigated in models of MS. This agent would be an excellent candidate as a new immunomodulatory agent since it exhibits minor side effects, especially in comparison to current immunosuppressive therapies.

Here, we focus on Gz-mediated immunomodulation of microglia and macrophages in two models of MS: EAE and cuprizone-induced demyelination. Microglia, the CNS-resident innate immune cells, play a key role in the pathogenesis of MS and their activation is considered a hallmark of the disease^11,12^. The EAE model, in particular, has been instrumental in shaping our understanding of microglia’s diverse roles within the inflamed CNS. There is evidence that microglia act as a driver of MS pathology: ablation of microglia suppresses EAE development^13,14^ and microglia in plaque regions of human brain samples express high levels of major histocompatibility class II (MHC II) molecules, suggestive of active antigen presentation to pathogenic T cells^15–17^. Blood-derived macrophages also enter the CNS due to compromised blood–brain barrier integrity in MS. Macrophages take on many of the same roles as microglia, however, some studies suggest that these infiltrating peripheral cells are more pathogenic in MS/EAE compared to resident microglia^18,19^. Understanding how Gz affects these two major cellular drivers of neuroinflammation is important to elucidate if this drug is to become clinically relevant for neurodegenerative and/or autoimmune diseases.

## Results

### Therapeutic Gz attenuates EAE symptoms

**Figure 1:**
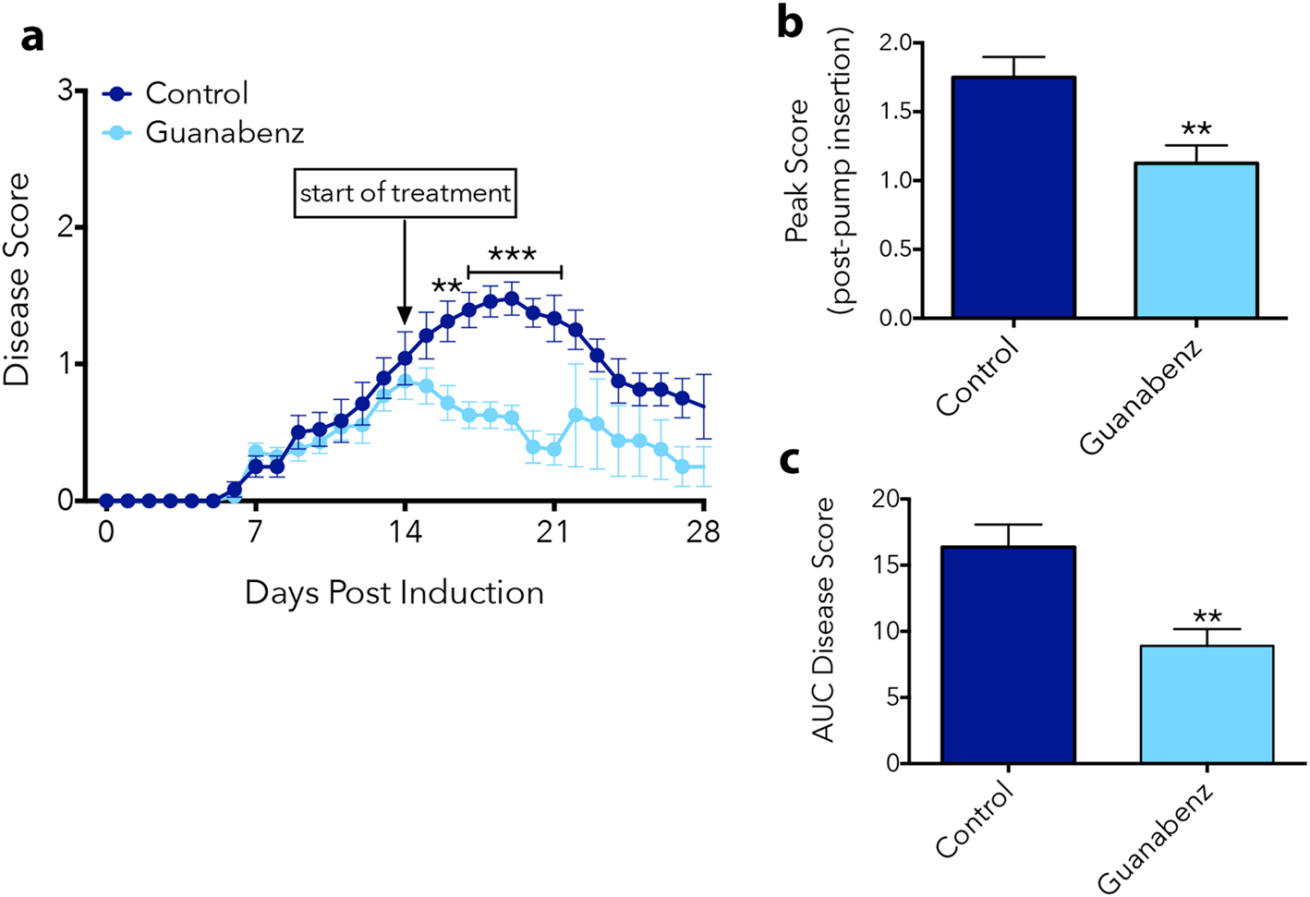
Therapeutic treatment with Gz attenuates EAE. MOG-EAE was induced in C57BL/6 female mice on Day 0 by subcutaneous injection of MOG_35-55_ in CFA and intraperitoneal injection of pertussis toxin on Days 0 and 2. Fourteen days post-MOG immunization, mini-osmotic pumps containing Gz (3 mg/kg/day) were implanted subcutaneously in the backs in a random subset of mice. Disease score was assessed blindly daily and animals were euthanized on Day 21 or Day 28 for histological analysis (**a**). Peak score indicates the highest score achieved by mice at time points after drug pump insertion (**b**). Area under the curve was calculated to measure overall illness throughout the course of the disease (**c**). Data are mean ± SEM. n = 14 control, 16 Gz-treated (D0-21), n = 4 (D21-28); Significantly (*p < 0.05, ***p < 0.001) different from control.

We first employed Gz in EAE models to investigate its potential to dampen inflammation within the CNS, since it has been shown to exert anti-inflammatory effects in other contexts^7–10^. Monophasic EAE was induced in 8–10-week-old female C57BL/6 mice using MOG_35-55_, and motor dysfunction was assessed daily using a disease score measure of 0 (no deficits) through 5 (death). Mini-osmotic pumps containing Gz were implanted subcutaneously on Day 14 post-MOG immunization to model a therapeutic timeline, where mice received constant infusion of the drug (3.0 mg/kg/day). Here, we observed that Gz dynamically attenuated EAE, reducing disease scores significantly at the peak of symptoms (Fig. 1a,b). Quantification of the area under the curve (AUC) indicated less overall behavioral signs throughout the course of the disease in Gz-treated mice (Fig. 1c). The effectiveness of Gz was also evaluated in the relapsing–remitting EAE model induced using PLP_139-151_ immunization. Gz infusion was initiated at Day 21 post-PLP immunization, as mice were recovering from an initial peak in symptoms. This proof-of-concept experiment suggests that Gz-infused animals exhibited less severe relapses (Figure S1).

### Therapeutic Gz reduces demyelination

**Figure 2:**
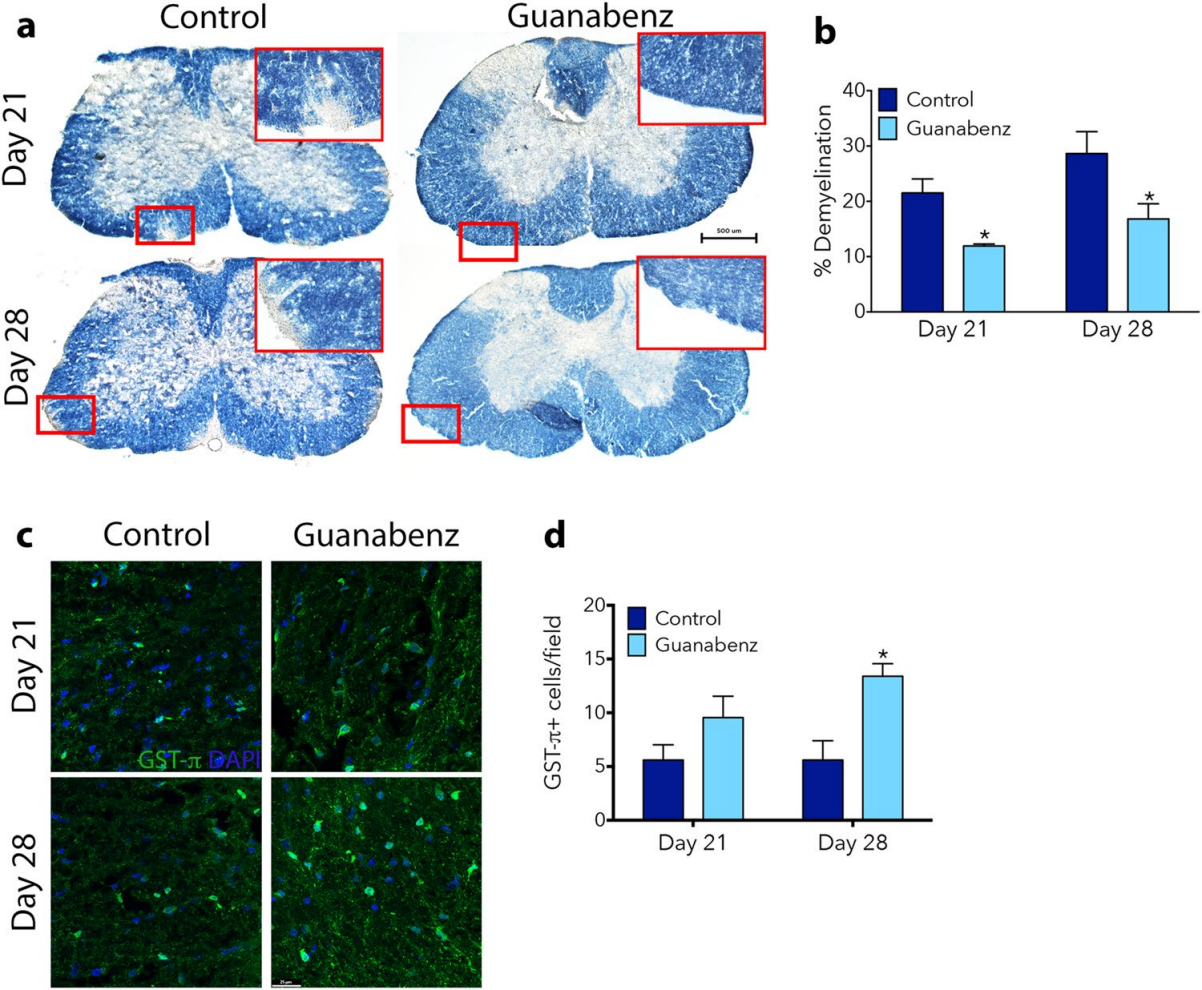
Gz reduces demyelination over the course of MOG-EAE. Lumbar spinal cord was isolated from control or Gz-treated mice at Day 21 or 28 post-induction of EAE. Frozen 20 μm sections previously stored on slides were stained with eriochrome cyanine (EC) to visualize myelin. Myelinated regions of white matter stain blue, whereas demyelinated regions are recognized by diminished color (shown in insets). Images of full coronal sections are shown in (**a**). Scale bar: 500 μm. Demyelinated areas were measured using thresholding in ImageJ and quantified as percent demyelination in (**b**). To visualize oligodendrocytes in the ventral white matter, where demyelination was evident, immunostaining for GSTπ (green) was performed. Cell nuclei were identified by DAPI (blue) (**c**). Scale bar: 25 μm. The number of GSTπ+ cells/field were quantified (**d**). Data are mean ± SEM. n = 3–6; Significantly (*p < 0.05) different from control.

The extent of demyelination was evaluated in the lumbar spinal cord after 7 (Day 21 post-EAE onset) and 14 (Day 28 post-EAE onset) days of therapeutic Gz infusion. Eriochrome cyanine (EC) staining (Fig. 2a) revealed a significant reduction in percent demyelination at both time points over the course of the disease (Fig. 2b). This is consistent with a previous report that initiated daily i.p. injections of Gz 7-days post MOG-immunization and observed a reduction in demyelination^6^. Way et al. attributed the reduced demyelination upon daily Gz injections to an improvement in oligodendrocyte survival in the inflamed CNS. This finding is also consistent with our results when we visualized mature oligodendrocytes in the ventral white matter areas where demyelination is observed (as indicated in Fig. 2a) with the marker GST-π (Fig. 2c,d).

### Gz alters microglia/macrophage numbers in EAE

**Figure 3:**
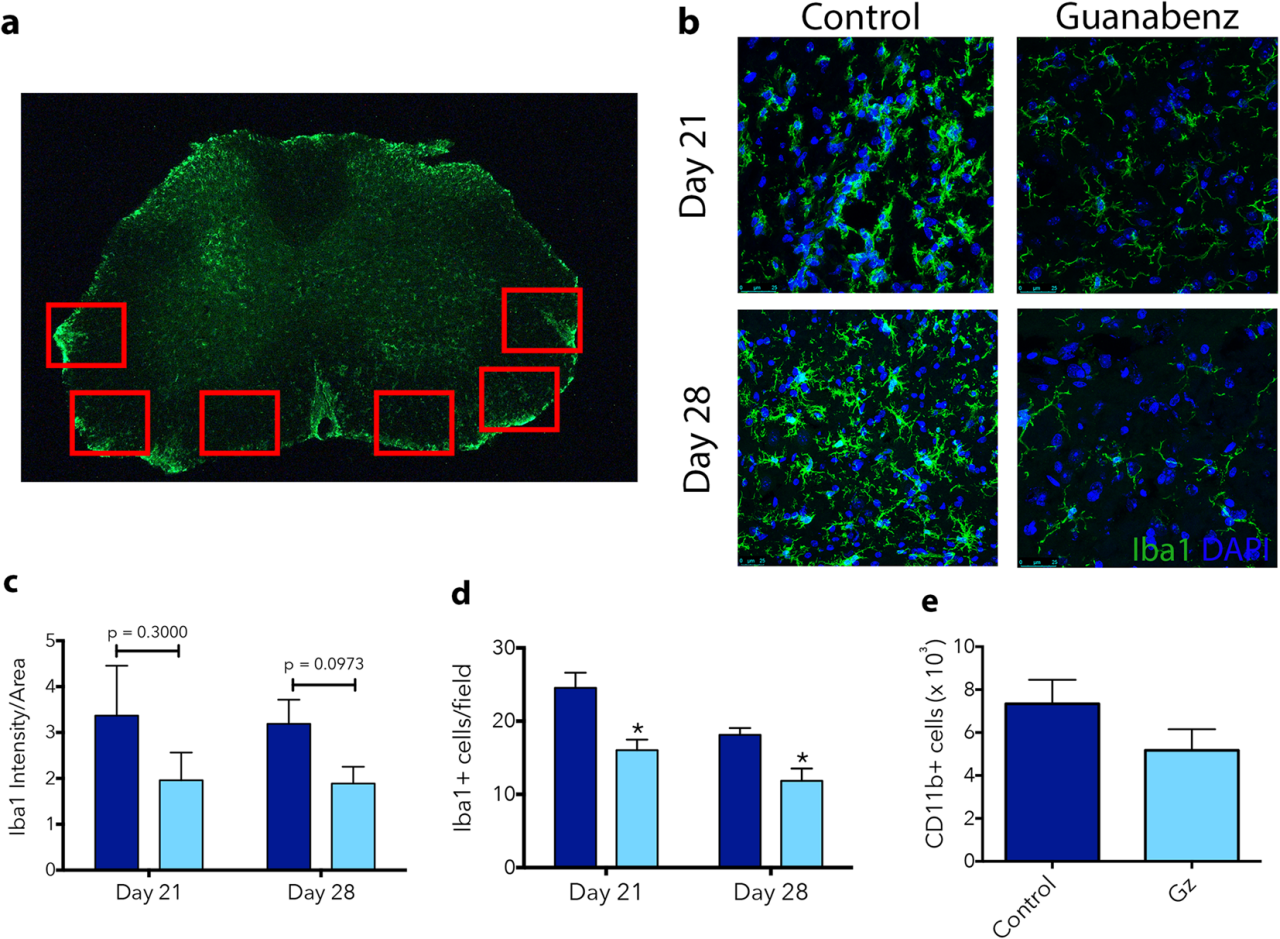
Gz reduces microglia/macrophage numbers in the lumbar spinal cord over the course of EAE. Frozen lumbar spinal cord sections isolated from EAE mice at Day 21 or 28 post-MOG immunization were immunofluorescently stained with Iba1 (green), to identify microglia and macrophages. Images of full coronal sections and the pre-designated locations where high magnification images were taken are shown (**a**). Scale bar: 500 μm. High magnification images used for cell counts are shown in (**b**). Iba1 fluorescence intensity was quantified using thresholding of the whole spinal cord images in ImageJ (**c**). Iba1+ cells per field of view were quantified from higher magnification images (**d**). For flow cytometric analysis, the spinal cord was isolated at Day 21 post-induction of MOG-EAE. Tissue was digested and a 30% Percoll centrifugation step was performed to remove myelin. The single cell suspension was then stained with CD11b to identify microglia/macrophages (**e**). Data are mean ± SEM. n = 3–5 (**a**–**d**), n = 4–6 (**e**).

For broad evaluation of microglia/macrophage activation in untreated and Gz-treated EAE animals, Iba1 immunostaining was performed on lumbar spinal cord sections at Day 21 and Day 28 post-MOG immunization, imaged on whole sections and its intensity quantified (Fig. 3a). Increase in Iba1 intensity is either a result of microglial proliferation and/or monocyte infiltration or is due to their activation^20^. Thus, it is indicative of a microglia or monocytic inflammatory response. Though not significant, there were reductions in Iba1 intensity in the whole spinal cords of Gz-treated animals throughout the course of EAE (Fig. 3c). We then evaluated the number of microglia/macrophages present by quantifying cells per field in high magnification images (Fig. 3b) from ventral white matter areas where inflammation and demyelination are most prominent (Fig. 3a, red boxes). Gz treatment resulted in a significant decrease in Iba1+ cells per field at both Days 21 and 28 post-MOG immunization (Fig. 3d). We also assessed the number of CD11b+ monocytes/macrophages in the spinal cord at Day 21 by flow cytometry and did not observe differences (Fig. 3e). Additionally, we probed astrocyte activation by assessing GFAP intensity and observed no differences (Figure S2).

### Gz-treated EAE mice exhibit reductions in iNOS+ and CD86+ microglia/macrophages

**Figure 4:**
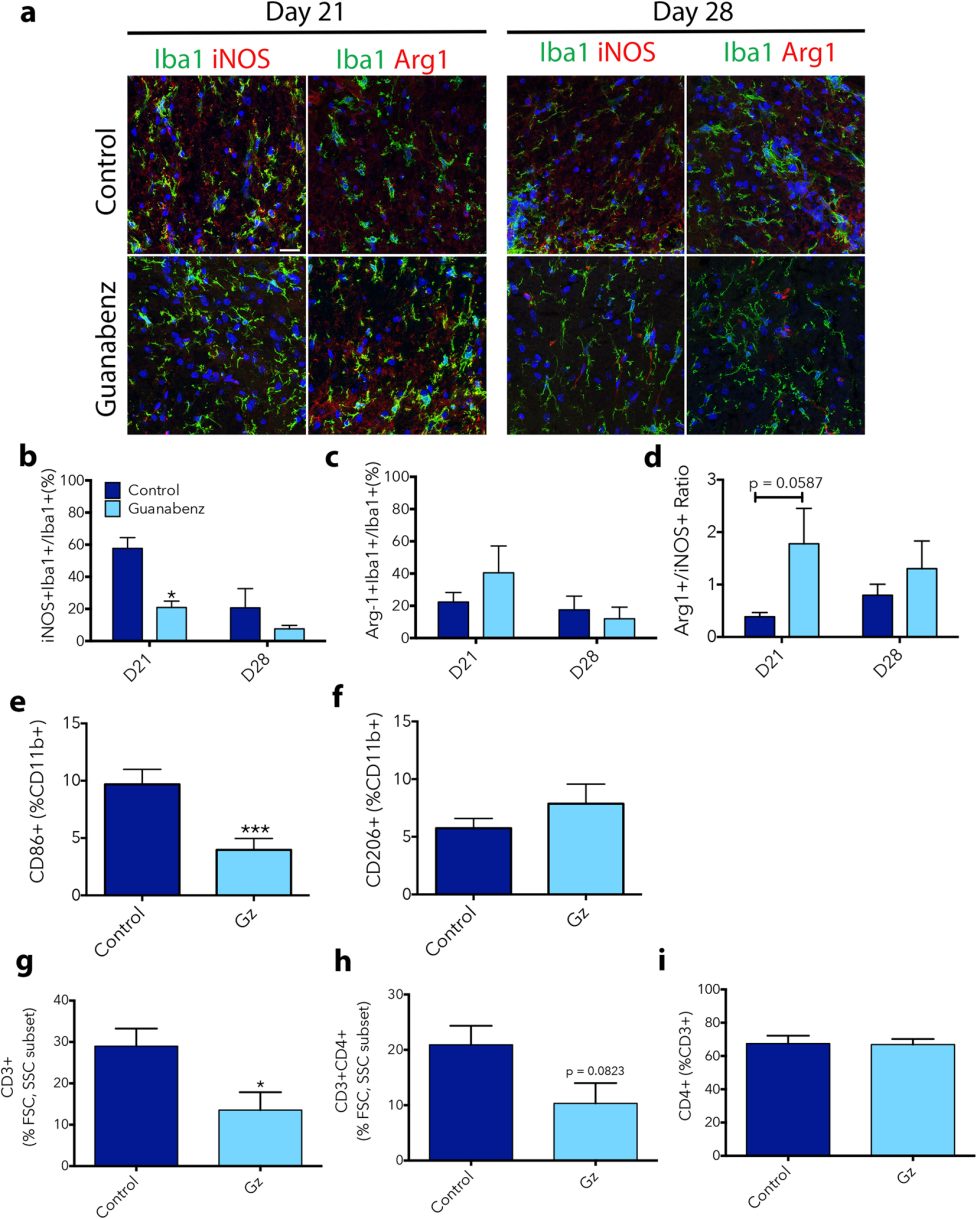
Gz reduces the expression of pro-inflammatory proteins on microglia/macrophages in EAE. Lumbar spinal cord sections were isolated from control or Gz-treated EAE animals at Day 21 or 28 post-disease induction. Pro-inflammatory microglia/macrophages were identified by immunofluorescent co-localization of the inflammation marker iNOS (red) with the general microglia/macrophages marker Iba1 (green). Anti-inflammatory microglia/macrophages were identified by co-localization of Arg1 (red) and Iba1 (green). Nuclei are identified by DAPI (blue) (**a**). Scale bar: 20 μm. iNOS+ Iba1+ or Arg1+ Iba1+ cells were counted and reported as a percentage over total Iba1+ cells (**b**) and (**c**), respectively. A ratio of Arg1+ to iNOS+ microglia/macrophages was calculated in (**d**). For flow cytometric analysis, the spinal cord was isolated at Day 21 post-induction of MOG-EAE. Tissue was digested and a 30% Percoll centrifugation step was performed to remove myelin. The single cell suspension was then stained with CD11b to identify microglia/macrophages (**e**) and (**f**), CD86 to delineate pro-inflammatory cells (**g**) and CD206 to mark anti-inflammatory cells (**h**). For T cell analysis, single cell suspensions from spinal cords isolated from EAE mice at Day 21 were stained with CD3 to identify all T cells and CD4 to mark CD4+ T cell subset. Analysis was performed on an LSR Fortessa. Post-processing was done on FlowJo software and data is reported as percentages. Data are mean ± SEM. n = 3–4 (**a**–**d**), n = 4–6 (**e**–**i**). Significantly (*p < 0.05, **p < 0.01) different from control.

Gz has previously been reported to reduce microglial numbers in the spinal cord in EAE^6^, but the pro- or anti-inflammatory status of these cells has not been assessed. We first used immunofluorescence to evaluate expression of pro- and anti-inflammatory markers over the course of EAE. iNOS was used as a pro-inflammatory marker, while Arg1 was employed as an anti-inflammatory marker (Fig. 4a). Significantly lower percentages of iNOS+ pro-inflammatory microglia/macrophages were evident in tissue from Gz-treated animals compared to control animals at Day 21, when inflammation was the most severe in controls (Fig. 4b). There were no significant differences in the percentage of Iba1+ cells expressing Arg1 at Day 21, or at Day 28 (Fig. 4c). When the ratio of Iba1+ Arg1+ to Iba1+ iNOS+ cells was calculated, a trend towards anti-inflammation was observed due to the decrease in iNOS+ cells; the difference was not statistically significant (Fig. 4d).

We also employed flow cytometry to probe a different set of pro- and anti-inflammatory markers in microglia/macrophage at Day 21. Spinal cords of control and Gz-treated EAE mice were isolated, digested, and stained with CD11b to identify the general microglia/macrophage population. CD86/B7.2, which participates in antigen presentation^21^, was used as a pro-inflammatory marker and CD206 was used as an anti-inflammatory marker. Gating strategy is shown in Figure S3. In agreement with our immunofluorescent results, the percent of pro-inflammatory microglia/macrophages (CD86+) was significantly decreased (Fig. 4e), whereas the percent of anti-inflammatory CD206+ cells was unchanged (Fig. 4f). Taken together, both immunofluorescent and flow cytometric analyses revealed that Gz treatment results in fewer microglia/macrophages expressing pro-inflammatory proteins at the Day 21 inflammatory peak of EAE.

### Gz reduces T cells in the spinal cord of EAE mice

T cells are central to the cellular pathology of MS/EAE and interact with both microglia and macrophages, responding to chemokines/cytokines and participating in antigen presentation. Further, previous studies have observed alterations in T cell numbers and function in response to Gz^6,10^. We used flow cytometry to quantify total T cells and CD4+ T cells in the spinal cord of EAE animals, since myelin damage in EAE has been attributed mainly to CD4+ T cells, rather than CD8+ T cells^22,23^. Gating strategy is shown in Figure S4. At Day 21, there was a significant decrease in the percent of CD3+ T cells in the spinal cord of Gz-treated animals compared to control animals (Figs. 4g, S5). There were no differences in the percent of CD3+ CD4+ T cells (Fig. 4h) nor in the percent of CD3+ T cells that were CD4+ positive (Fig. 4i) in the spinal cords of control or Gz-treated animals.

### Gz directly alters microglia function

**Figure 5:**
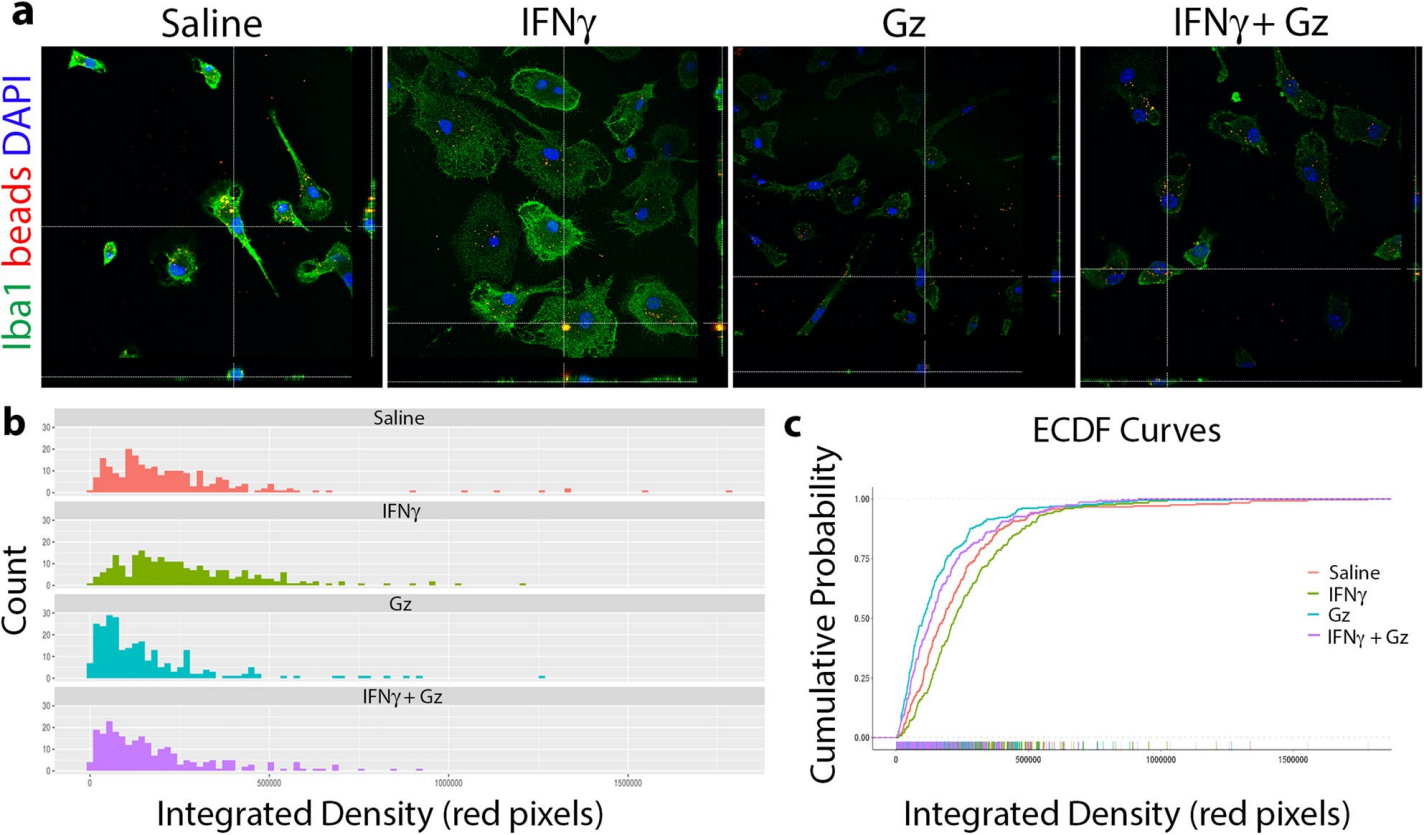
Gz diminishes phagocytic activity of primary microglia. Microglia were isolated from mixed cortical cultures derived from P0-2 C57BL/6 mice. Cells were treated with saline, 100 U/µl IFNγ, 10 µm Gz, or both IFNγ and Gz for 24 h. 0.5 µm latex beads (red) were then added to the cultures for 2 h prior to fixation and staining of the cells with Iba1 (green). DAPI (blue) was used to identify cell nuclei (**a**). The integrated density of red pixels (beads) per cell was measured on ImageJ for a total of 238–260 cells from 3 independent experiments (**b**). The integrated density data was then uploaded to Github (https://github.com/mcap91/Monocyte-Phagocytosis-Assay) to generate ECDF curves (**c**) and analyzed using a K-S test.

To assess whether the observed Gz-mediated immunomodulatory effects in EAE are due to direct alterations of function, we directly exposed primary microglia to Gz and examined phagocytosis, a primary function of microglia that precedes antigen presentation to T cells^24,25^. To assess whether Gz affected the phagocytic capabilities of microglia, cells were stimulated with interferon-gamma (IFNγ), an MS-relevant cytokine secreted by T cells that infiltrate the CNS in MS/EAE^26^. IFNγ stimulates an increase in phagocytic activity of microglia in vitro^27^. Cells were incubated with saline (vehicle control), IFNγ, Gz, or both IFNγ and Gz in the presence of fluorescent latex beads (Fig. 5a). As expected, IFNγ increased phagocytic activity compared to saline. Gz treatment alone decreased the phagocytic activity of microglia compared to both saline-treated microglia and IFNγ-stimulated cells. Importantly, when the cells were treated with both IFNγ and Gz, phagocytic activity was decreased compared to both saline-treated and IFNγ-stimulated microglia (Fig. 5b,c, Table S1). Additionally, iNOS expression was assessed in Gz-treated primary microglia and surprisingly, no notable decreases were evident in the percentage of IFNγ-stimulated microglia expressing iNOS when treated with Gz, compared to cells only treated with IFNγ (Figure S6).

### Gz does not improve outcomes in the cuprizone model of demyelination

**Figure 6:**
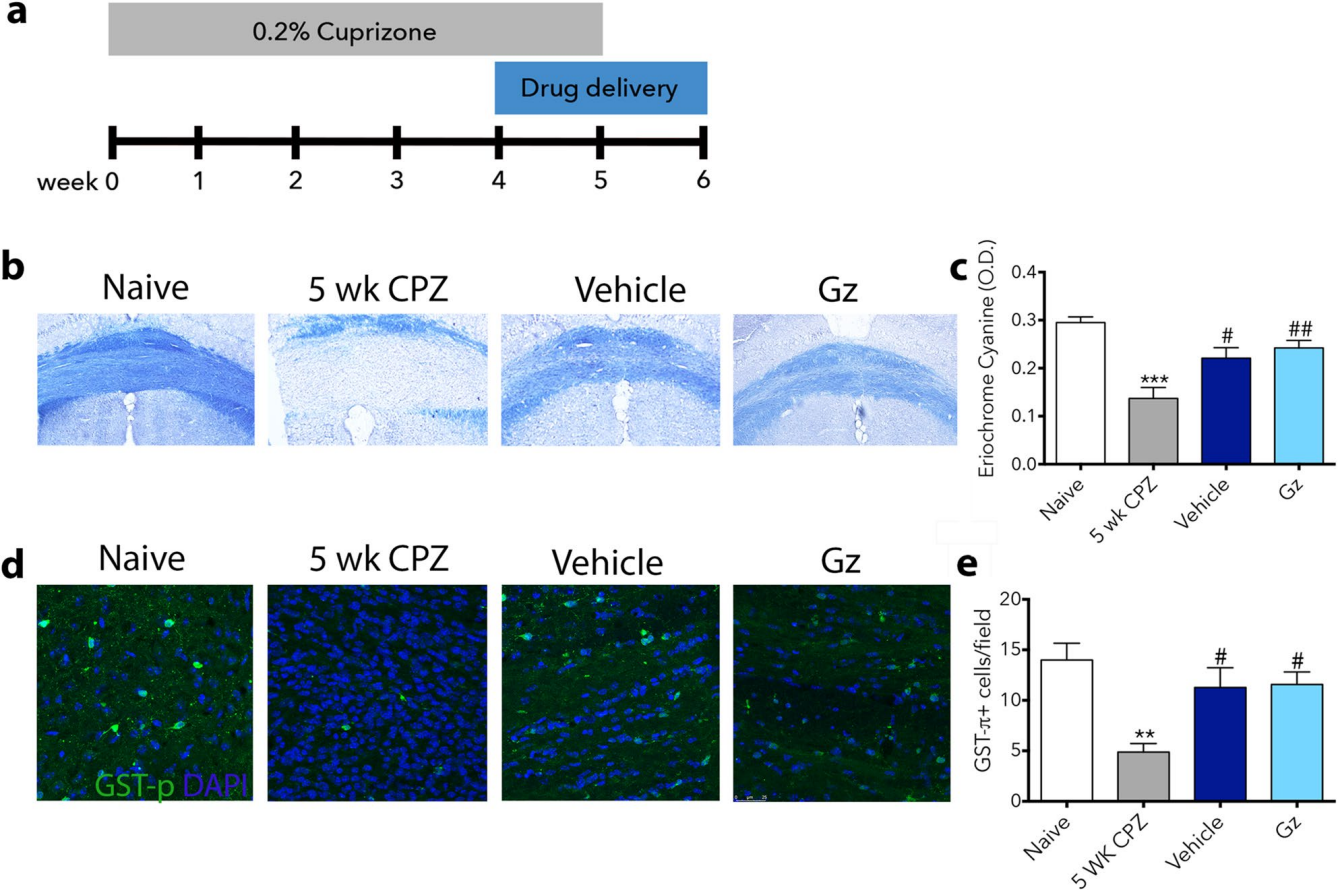
Gz does not significantly accelerate remyelination in the cuprizone model of demyelination. Mice were fed 0.2% cuprizone in their chow for 5 weeks. At week 4, mini-osmotic pumps containing saline (vehicle) or Gz were implanted subcutaneously. Mice were reverted to standard chow for 1 week to allow for spontaneous remyelination (**a**). At 5 weeks, before the withdrawal of cuprizone, both naïve mice and cuprizone-fed mice were euthanized for baseline analysis to assure demyelination in the corpus callosum, using EC staining. After 1 week of cuprizone withdrawal, vehicle and Gz-treated mice were collected and EC stain was used to visualize remyelination in the corpus callosum (**b**). The optical density of EC was calculated using ImageJ (**c**). Data are mean ± SEM. n = 4–6. ***p < 0.05 compared to naïve, ^#^p < 0.05, ^##^p < 0.01 compared to 5 week CPZ.

Gz has been previously shown to affect oligodendrocyte survival^6^, and our data indicate that it affects the pro-inflammatory state of microglia/macrophages in inflammatory demyelination. To dissect whether immune effects or oligodendrocyte survival dominates its beneficial effects, we evaluated Gz in a non-immune mediated model of demyelination, using cuprizone, which is selectively and reversibly toxic to oligodendrocytes^28^. Cuprizone was administered in mouse chow for 5 weeks and a subset of mice was collected at week 5 to confirm demyelination. Gz administration was initiated at week 4 of cuprizone intoxication and continued for 1 week after cuprizone withdrawal (Fig. 6a). There was significant demyelination after 5 weeks of cuprizone administration compared to naïve mice. After 1 week of remyelination, both vehicle and Gz-treated animals displayed significantly more myelin in the corpus callosum compared to the 5-week baseline. However, vehicle and Gz-treated animals were not significantly different from one another (Fig. 6b,c).

The number of oligodendrocytes present in the corpus callosum was consistent with the myelin analysis. Using GSTπ immunofluorescence to quantify mature oligodendrocytes (Fig. 6d), a significant decrease in GSTπ+ cells was observed in cuprizone-fed mice compared to control. One week after cuprizone withdrawal (and return to normal chow), there were significant increases in the number of oligodendrocytes in both vehicle and Gz-treated animals compared to the 5-week cuprizone-fed mice. There were no differences between the treatment groups (Fig. 6e).

**Figure 7:**
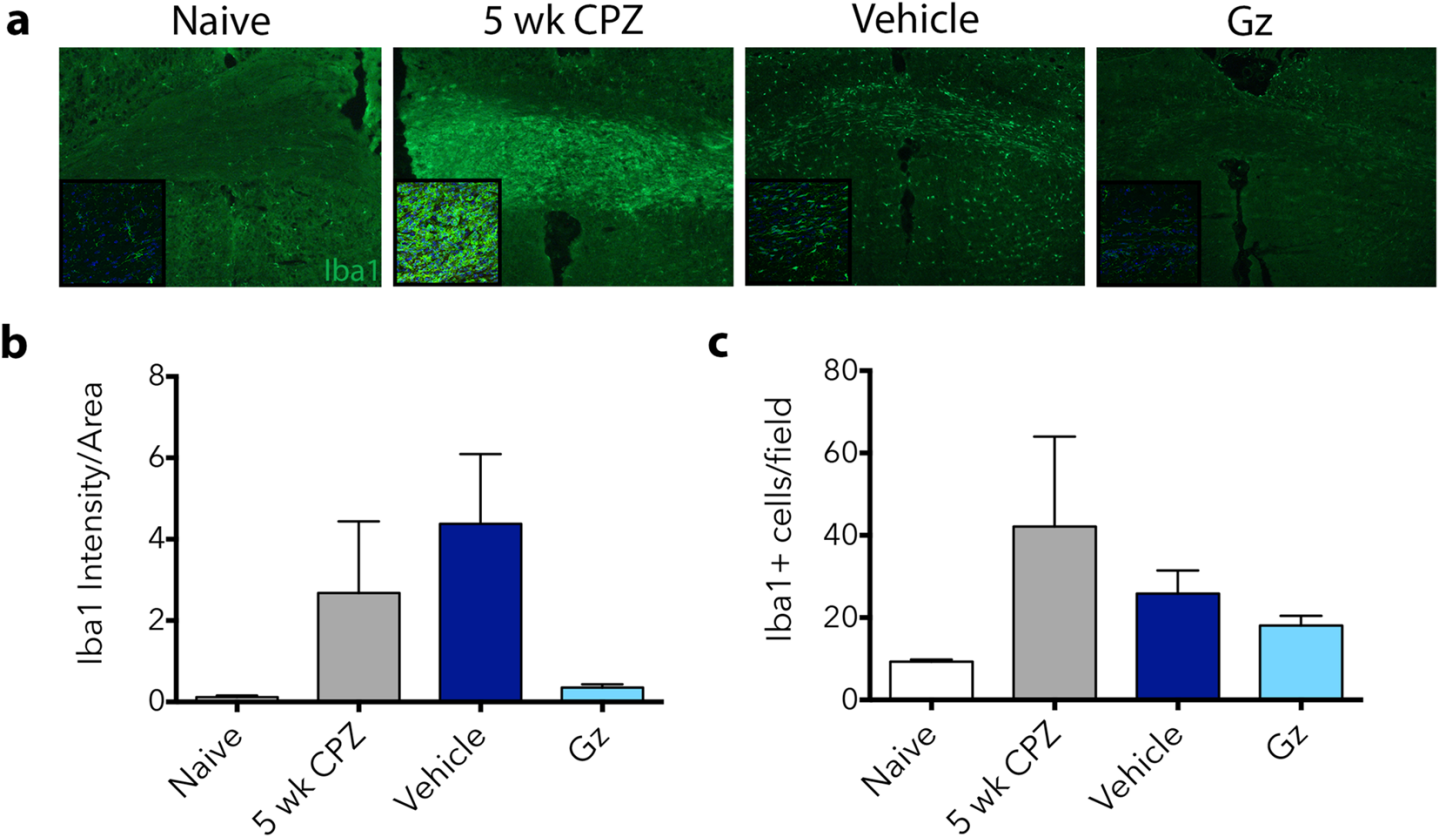
Gz does not dramatically affect microglial activation in cuprizone-fed mice. Mice were fed 0.2% cuprizone in their chow for 5 weeks. At week 4, mini-osmotic pumps containing saline (vehicle) or Gz were implanted subcutaneously. Mice were reverted to standard chow for 1 week to allow for spontaneous remyelination. At 5 weeks, before the withdrawal of cuprizone, both naïve mice and cuprizone-fed mice were euthanized for baseline analysis to assure microglial activation by Iba1 immunofluorescent staining (green). After 1 week of cuprizone withdrawal, vehicle and Gz-treated mice were collected Iba1 staining performed (**a**). Iba1 intensity was calculated using ImageJ (**b**). Cells per field were counted manually in high magnification images (insets in **a**,**c**) Data are mean ± SEM. n = 4–6.

It was recently reported that microglia, but not infiltrating macrophages, are found in cuprizone lesions, and that microglia are required for cuprizone-mediated demyelination^29^. When we assessed the activation and number of microglia in the corpus callosum, Gz treatment resulted in decrease of the intensity of Iba1 fluorescence and the number of Iba1+ cells per field, though there was variability in cuprizone-fed and vehicle-treated animals (Fig. 7).

## Discussion

Gz was previously reported to improve EAE through enhancing the ISR in oligodendrocytes, thus improving their survival and remyelination in the inflamed CNS^6^. Here, we further characterize the beneficial actions of Gz, showing that amelioration of EAE can also be attributed to dampening the pro-inflammatory responses of microglia and macrophages. Distinctly from the study by Way et al. which first employed Gz in EAE via daily i.p. injections starting on Day 7 post-MOG immunization, we started treatment later, at Day 14, by infusion with mini-osmotic pumps that were surgically implanted subcutaneously in the backs of EAE mice. With this more relevant therapeutic timescale and continuous infusion of drug, we did observe decreased demyelination, increased oligodendrocyte cell numbers, and decreased microglia/macrophages and T cells in the spinal cord in agreement with Way et al.

The immunomodulatory activity of Gz was examined in detail: dramatic reductions in the percent of microglia/macrophages expressing pro-inflammatory markers were observed at Day 21 post-MOG immunization, the time point of peak inflammation. Immunofluorescent analysis showed a significant decrease in iNOS expression and flow cytometric analysis revealed a significant decrease in expression of CD86 on microglia/macrophages. During homeostasis, CD86 and other molecules involved in antigen presentation are barely detectable in microglia, but become upregulated during inflammation. In the context of EAE, it has been reported that the first step of microglial activation is upregulation of MHCII, CD40, and CD86, which provide co-stimulatory signals required for T cell activation^30^. The Gz-mediated decrease in the percentage of microglia/macrophages expressing CD86 could potentially indicate the inability of microglia to successfully activate autoreactive T cells.

It is possible that the enhancement of the ISR in oligodendrocytes, which reduces oligodendrocyte cell death, is responsible for decreased demyelination and the improvements in motor deficits in the spinal cord, as previously reported^6^. We confirmed an increase in the number of mature oligodendrocytes in the spinal cord in addition to the decrease in pro-inflammatory microglia. Importantly, however, we show that Gz diminishes phagocytic activity of both unstimulated and IFNγ-stimulated microglia in vitro. This is a notable finding as it demonstrates that Gz is directly influencing microglial function upon stimulation with an MS-relevant pro-inflammatory cytokine. Though phagocytosis is generally considered a beneficial function of microglia in EAE as myelin debris is deemed an obstacle to myelin repair^31,32^, the uptake of antigens via phagocytosis precedes antigen presentation to encephalotigenic T cells. Thus, this process of phagocytosis can also promote CNS inflammation. A study of the kinetics of myelin uptake by microglia and other antigen-presenting cells (APCs) in EAE reported that APCs that had taken up myelin antigen were often co-localized with CD4+ T cells, indicative of active antigen presentation^33^. Another study found that microglia are also able to take up neuronal antigens, such as NF-L. They suggested that though the clearance of neuronal debris is necessary, this process may amplify autoimmunity by the presentation of these neuronal antigens to T cells^25^. Therefore, this direct functional impairment of microglia by Gz could also result in the observed decreases in T cells in the spinal cord (Fig. 4g). It is also possible that other antigen-presenting cells that we have not examined, such as B cells, perivascular macrophages, and dendritic cells, are modulated by guanabenz. Though Way et al. did not find any differences in B cell or dendritic cell numbers in the spinal cord of EAE animals treated with guanabenz compared to controls^6^, other studies have shown that guanabenz inhibits activation of both cell types^8,9^.

We further assessed the effectiveness of Gz in a toxin-induced model of demyelination to contrast the severe CNS inflammation in EAE. The cuprizone model, which induces selective oligodendrocyte cell death^28,34^, was chosen as there is evidence that oligodendrocyte viability during exposure to cuprizone is dependent on the ISR pathway^35,36^. Surprisingly, we did not observe a significant increase in oligodendrocyte numbers in Gz-treated mice compared to saline-treated animals after the withdrawal of cuprizone (but continuous infusion/presence of Gz). This could mean that dampening the immune response in the more inflammatory setting of EAE is the more predominant mechanism attenuating disease. In the cuprizone model, a decrease in the intensity of Iba1 (an indicator of microglial activation) was evident, but it was not significant. A recent study reported that microglia, not peripherally-derived macrophages, dominate cuprizone lesions and that microglial activation is sufficient to drive demyelination even in the absence of cuprizone^29^. It is possible that Gz-mediated differences in microglial activation are apparent at the early timepoints during active demyelination, but these effects were no longer dramatic at the time points assessed in this study. Further, initiating Gz infusion earlier than week 4 of cuprizone intoxication may be more beneficial, as other studies have shown that there is a window to induce microglia-mediated benefits, with positive effects being observed when agents are administered earlier during cuprizone intoxication^37^.

It will be important to further understand the inflammation-related signaling pathways mediated by Gz. Several reports have suggested starting points such as α2-adrenergic agonism^38^, elevation of eIF2α phosphorylation^39^, and a decrease in cholesterol hydroxylase^9^, all of which intersect with immune pathways in microglia and/or macrophages.

In conclusion, our results indicate that Gz significantly dampens the pro-inflammatory responses of microglia and macrophages in EAE, and results in decrease of the number of T cells, the main pathogenic cell type in this model. As there have been several recent reports of anti-inflammatory properties of Gz in other disease models, it is important to characterize the beneficial immunomodulatory actions in contexts in which this drug could potentially be repurposed in the clinic.

## Methods

### Animals

Wild-type female C57BL/6 (Jackson Laboratory) or SJL/J mice (Charles River) were bred in-house under specific pathogen-free conditions set by Stony Brook University Division of Laboratory Animal Resources (DLAR). Mice were provided food and water ad libitum and were maintained on a 12-h light/dark cycle. All procedures were approved by the Stony Brook University Institutional Animal Care and Use Committee (IACUC) and all experiments and methods were performed in accordance with the IACUC-approved procedures.

### Induction of monophasic or relapsing–remitting EAE

Monophasic EAE was induced in 8–10-week-old female C57BL/6 mice by subcutaneous injection of 300 µg of MOG_35-55_ peptide (Biomatik, USA, LLC; Sequence: MEVGWYRSPFSRVVHLYRNGK) emulsified in Complete Freund’s Adjuvant (CFA) containing 500 µg of heat-inactivated Mycobacterium tuberculosis (Difco). On Days 0 and 2, 500 ng pertussis toxin (List Biologicals) was administered intraperitoneally. Drug treatment was initiated on Day 14 post-disease induction. To induce relapsing–remitting EAE, injection of 200 µg of PLP_137-151_ peptide (Biomatik, USA, LLC; Sequence: HSLGTKWLGHPDKF) emulsified in CFA containing 4 mg/mL heat-inactivated Mycobacterium tuberculosis (Difco) was administered subcutaneously in 8–10-week-old SJL/J mice, as previously described^40^. Drug treatment was initiated on Day 21 post-disease induction. For both monophasic and relapsing–remitting EAE, mice were weighed weekly and observed daily to evaluate symptom severity. Symptoms were scored in a blind manner on a scale of 0–5 with gradations of 0.5 for intermediate symptoms. Scores are as follows: 0 = no detectable symptoms, 1 = loss of tail tone, 2 = abnormal gait, 3 = hindlimb paralysis, 4 = forelimb weakness/paralysis, 5 = moribund/dead.

### Cuprizone-induced demyelination

Wild-type 8–10-week-old female C57BL/6 mice were fed 0.2% (w/w) cuprizone mixed into standard rodent chow (Envigo). The mice were maintained on the cuprizone diet for 5 weeks at which point they were reverted to standard chow. A subset of naïve and cuprizone-fed untreated mice was euthanized at week 5 to validate demyelination. One week prior to the withdrawal of cuprizone, vehicle or Gz treatment was initiated. One week post-cuprizone withdrawal, vehicle or Gz-treated mice were euthanized and brains were isolated and processed as described below.

### Drug delivery

Alzet mini-osmotic pumps (Durect) were used to deliver drugs in a time-controlled manner. For both EAE and cuprizone experiments, fourteen-day pumps (rate of infusion 0.25 µL/h, 100 µL total volume) were filled with Gz, and incubated overnight in sterile saline at 37 °C in a dry incubator before use. Pumps were implanted subcutaneously in the backs of anesthetized mice.

### Tissue processing for immunohistochemistry/immunofluorescence

Mice were deeply anesthetized by intraperitoneal injection of 2.5% avertin (0.2 mL/g body weight). Transcardial perfusion was performed using PBS, followed by 4% paraformaldehyde (PFA) in PBS (pH 7.4). Tissues were isolated and post-fixed in 4% PFA at 4 °C overnight. To cryopreserve, tissues were then transferred to 30% sucrose in PBS at 4 °C until no longer floating. For spinal cord, meningeal layers were removed under a dissecting microscope and the lumbar region was cut into equal sections prior to embedding in optimal cutting temperature compound (Tissue Tek, Sakura) and freezing at − 80 °C overnight to prepare for sectioning. For brains, cerebellum was removed prior to embedding and freezing as described above. 20 micron sections were obtained using a cryostat (Leica), placed onto slides, and stored at − 80 °C until use.

### Eriochrome cyanine staining

Eriochrome cyanine (EC) staining was used to visualize myelin in the lumbar spinal cord for EAE animals and in the corpus callosum of cuprizone-treated animals, as previously described^20,41,42^. Tissue sections stored on slides at − 80 °C were air-dried at room temperature overnight before incubation at 37 °C for two hours in a dry incubator. Slides were then submerged in acetone for 5 min and air-dried at room temperature for 30 min. The sections were stained in EC solution (0.2% EC (Sigma), 0.5% H_2_SO_4_ (Sigma), 10% iron alum (Sigma) in distilled water) for 30 min. Differentiation was achieved by exposing sections to 5% iron alum (Sigma) for 10 min, followed by borax-ferricyanide solution (1% borax (Sigma), 1.25% potassium ferricyanide (Sigma) in distilled water) for 5 min. Sections were dehydrated through graded ethanol solutions and coverslipped using SecureMount (Fisher Scientific). Stained sections were imaged on a Nikon Eclipse E600 microscope at 40× magnification. Images were then cropped to remove gray matter areas and FIJI freeware (NIH) was used to measure the demyelinated and total white matter area. Thresholding was used to obtain a binary signal that distinguishes between positive-staining white matter and negative-staining demyelination and percent demyelination was calculated as follows: Demyelinated area (%) = [(Demyelinated area in white matter/Total white matter area) × 100]. Six coronal spinal cord sections were analyzed for each biological replicate in EAE experiments and three full coronal brain sections were analyzed for cuprizone experiments.

### Immunofluorescence

Spinal cord or brain sections mounted on slides used for immunofluorescence were rinsed in PBS for 5 min to remove residual optimal cutting temperature compound. For in vitro experiments, cells plated on coverslips were fixed in 4% PFA for 20 min at room temperature and rinsed in PBS prior to staining. Samples were then blocked in the serum of the host of the secondary antibody (5% serum, 0.2% Triton X-100 in PBS) and incubated overnight at 4 °C with various combinations of primary antibody in 30 mg/mL BSA in PBS with 0.2% Triton X-100. Primary antibodies used include: rabbit anti-mouse Iba1 (1:500, Wako), mouse anti-mouse iNOS (1:500, BD Biosciences), mouse anti-mouse Arginase-1 (1:500, BD Biosciences), rabbit anti-mouse GSTπ (1:250, MBL International), and rabbit anti-mouse GFAP (1:500, Dako). After washing with PBS, sections were incubated with AlexaFluor 488- or 555-conjugated goat anti-rabbit or goat anti-mouse antibody for 2 h at room temperature. After rinsing with PBS, slides were mounted using Fluormount-G with DAPI (Southern Biotech). The sections were imaged using a Nikon Eclipse E600 microscope or a Leica Sp8X confocal microscope. Confocal images were acquired at the same pre-designated locations (indicated in Fig. 3) along the ventral columns of the lumbar spinal cord or the medial corpus callosum (cryostat sections taken between bregma − 0.7 through − 1.46) for each biological replicate^41^.

### Flow cytometry

Mice were deeply anesthetized by intraperitoneal injection of 2.5% avertin (0.2 mL/g body weight) and transcardially perfused with 50 mL of ice cold sterile PBS (pH 7.4). Whole spinal cord was isolated and digested in 10 mg/mL papain (Sigma) for 20 min with manual inversion every 5 min. Tissue was then triturated with a p1000 pipet and centrifuged for 5 min at 400*g* and 4 °C to remove papain solution. The pelleted cells were then resuspended in 30% Percoll (Sigma) in sterile Hank’s Balanced Saline Solution (HBSS). Samples were centrifuged for 15 min at 400*g* and 4 °C and the white myelin layer was carefully removed. The remaining Percoll solution containing the cells was diluted with sterile HBSS and centrifuged for 20 min at 400*g* and 4 °C to pellet the cells. After removal of the diluted Percoll solution, the cells were resuspended in FACS buffer (0.5% bovine serum albumin (BSA) in 1× sterile PBS) and passed through a 40-micron filter. Cells were counted using trypan blue exclusion. Cells were then blocked using anti-CD16/32 (1:50 in FACS buffer; Biolegend) for 30 min on ice and then stained with CD11b-APC, CD86-PE, CD206-Pacific Blue, CD3-Pacific Blue, CD4-PE antibodies (1:100 in FACS buffer; Biolegend) in various combinations for 30 min on ice in the dark. The staining solution was removed by centrifugation for 5 min at 200*g*, and cells were washed twice with FACS buffer before resuspending for flow cytometric analysis on a BD LSR Fortessa. Post-processing was performed on FlowJo software.

### Primary microglia culture

Primary microglia were harvested from the cortices of P0-2 C57BL/6 mice as previously described^42^. Briefly, after isolation of cortices, meninges and hippocampi were removed and the remaining tissue was digested in 0.25% Trypsin/EDTA (Sigma) at 37 °C/5% CO_2_ for 20 min. The tissue was then triturated and the Trypsin was neutralized with DMEM containing 10% FBS. Cells were pelleted by centrifugation and resuspended in mixed cortical culture medium (DMEM, 10% FBS, 1X antibiotic/antimycotic, and 40 μg/mL gentamicin). The cell solution was then filtered through a 40-micron cell strainer and plated in mixed cortical culture medium on plates that had been coated with poly-d-lysine (PDL, Sigma). Medium was changed 3 days later and microglia were harvested between Day 10 and 14.

To isolate the microglia from the mixed cortical culture, lidocaine was added at a final concentration of 1 mM and the culture was incubated at room temperature for 15 min. The medium, containing the floating microglia, was then centrifuged at 500*g* for 5 min, supernatant removed, and the cell pellet was resuspended in microglia medium (DMEM, 1% FBS, 1X antibiotic/antimycotic, 40 μg/mL gentamicin). Cells were counted on a hemocytometer before seeding into wells for experiments.

### Phagocytosis assay

Primary microglia isolated from C57BL/6 mixed cortical cultures as described above were plated on glass coverslips. After 24 h, cells were treated with either saline as a vehicle control, 100 units/mL IFN-γ (Roche), 10 μM Gz (Sigma), or both IFN-γ and Gz concomitantly. After 24 h of treatment, half of the medium was replaced with fresh medium containing rhodamine-conjugated latex beads (0.8 μm diameter, Sigma) at concentration of 0.1 μL bead suspension/mL medium. Cells were incubated at 37 °C/5% CO_2_ for 2 h after which non-internalized beads were thoroughly washed away with 1X HBSS. Cells were then fixed in 4% PFA for 20 min, rinsed with 1X PBS and immunofluorescently stained with rabbit anti-mouse Iba1 (Wako; 1:1000) and anti-rabbit AlexaFluor-488 before being mounted on slides with DAPI Fluormount (Southern Biotech). Microglia were imaged using a Leica SP8X confocal microscope. 10 μm z-stacks were taken at 40× resolution. Images were processed using Fiji^43^, where the integrated density of the sum-projected red pixels (beads) was measured.

### Statistical analysis

Figures were generated and statistical analysis was performed using Prism 6 software. For multiple comparisons within a group, one-way ANOVA followed by Tukey’s post-hoc test was used. For comparisons between two groups, two-tailed t-tests were employed. p < 0.05 was considered significant. For all figures, results are expressed as mean, with error bars representing the standard error of the mean. In all in vivo experiments, n refers to the number of biological replicates. For EAE scores, a non-parametric Mann–Whitney U test was used. For the phagocytosis assay, integrated density data was analyzed using a K-S test via GitHub (https://github.com/mcap91/Monocyte-Phagocytosis-Assay)^44^ and n refers to the total number of cells analyzed per treatment.

## Supplementary information

Supplementary Information.

## Data Availability

The datasets generated during and/or analyzed during the current study are available from the corresponding author on reasonable request.
