# Supplementary material for: Guanabenz modulates microglia and macrophages during demyelination
Source: Sci Rep. 2020 Nov 9;10:19333. doi: 10.1038/s41598-020-76383-w (PMC7653931; doi:10.1038/s41598-020-76383-w)
Supplement: Supplementary file 1 — Supplementary Information. [file 41598_2020_76383_MOESM1_ESM.docx]

**Guanabenz modulates microglia and macrophages during demyelination**

Kaitlyn Koenig Thompson^1^ and Stella E. Tsirka^1,^*

^1^Department of Pharmacological Sciences, Stony Brook University, Stony Brook, NY 11794, USA

*Correspondence: styliani-anna.tsirka@stonybrook.edu

**Supplementary Figure S1. Guanabenz improves disease score in a model of relapsing-remitting MS.** PLP-EAE was induced in 8-10-week-old female SJL/J mice on Day 0 by subcutaneous injection of PLP_137-151_ in CFA. Twenty-one days post-PLP injection (as mice recover from the first peak in symptoms), mini-osmotic pumps containing saline or guanabenz were implanted. Disease score was assessed daily and animals were euthanized on Day 49. Data are mean ± SEM. n = 2 saline-, 3 guanabenz-treated animals.

**
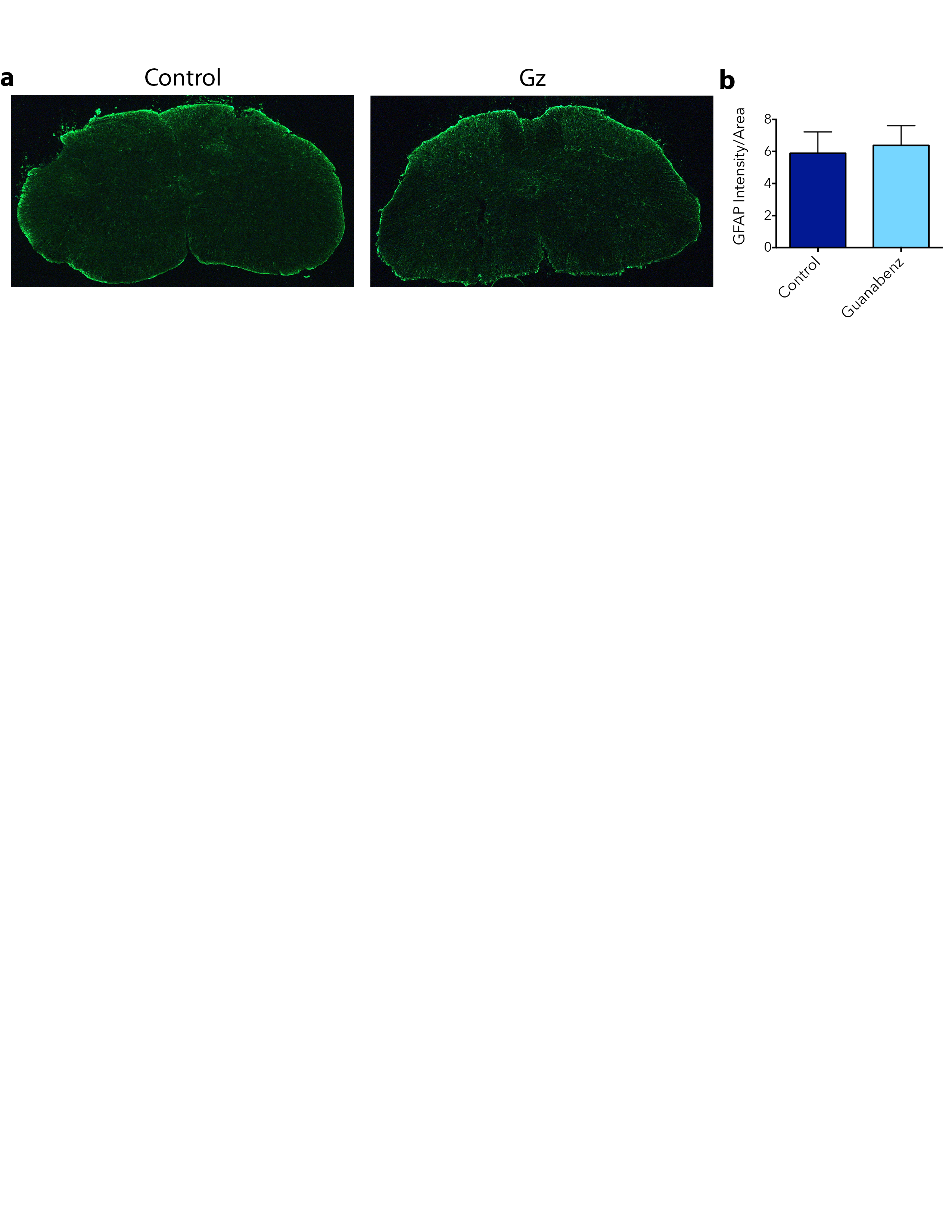
**

**Figure S2. Guanabenz does not alter astrocyte activation in the spinal cord at Day 21 post-MOG immunization.** Lumbar spinal cord sections from control or Gz-treated EAE mice at Day 21 were isolated and immunofluorescent stained for GFAP (green). Nuclei are identified by DAPI (blue) (**A**). GFAP fluorescence intensity was measured using thresholding in ImageJ (**B**). Data are mean ± SEM. n = 3-4.

**
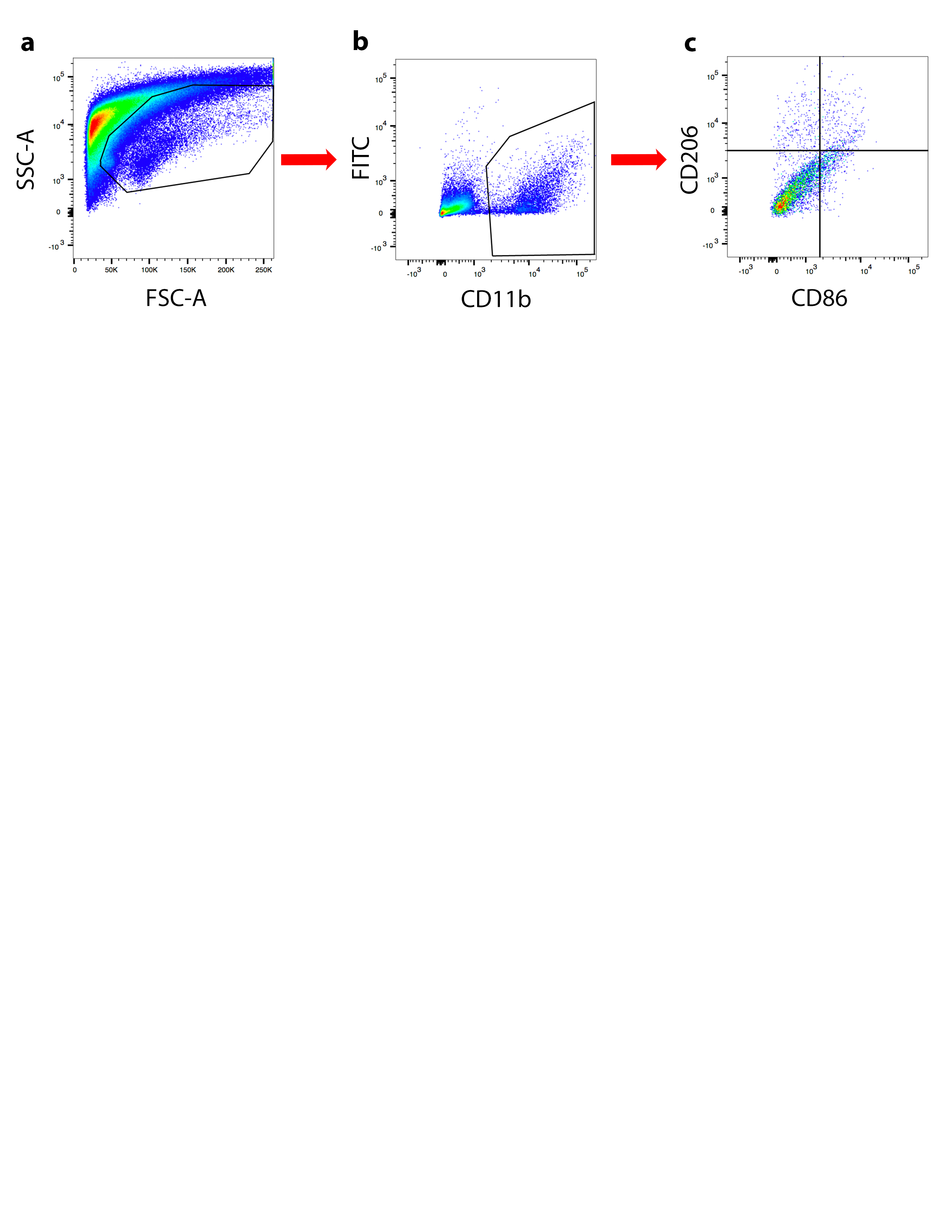
**

**Figure S3. Representative flow cytometry gating strategy for microglia/macrophages.** Spinal cords isolated from control or Gz-treated EAE mice were isolated at Day 21 post-MOG immunization. Tissue was digested, myelin removed by Percoll centrifugation, and the resulting single cell suspension was stained with CD11b, CD86, and CD206. FSC and SSC gating obtained was used to identify mononuclear cells based upon size and granularity (**A**), CD11b was used to obtain the microglia/macrophage population (**B**), and expression of CD86 and CD206 on CD11b+ cells was quantified (**C**).

**
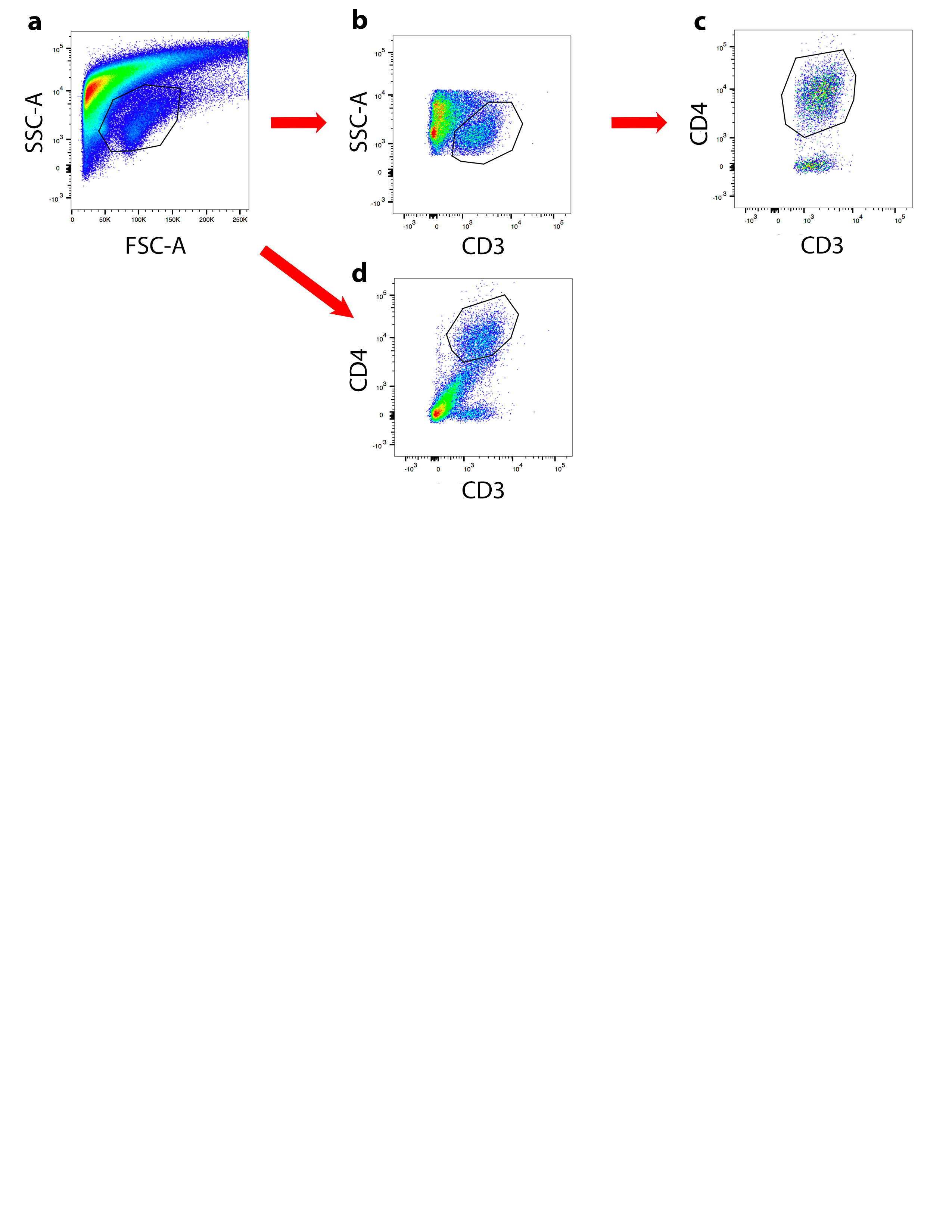
**

**Figure S4. Representative flow cytometry gating strategy for T cells.** Spinal cords isolated from control or Gz-treated EAE mice were isolated at Day 21 post-MOG immunization. Tissue was digested, myelin removed by Percoll centrifugation, and the single cell suspension was stained with CD3 and CD4. FSC and SSC gating obtained was used to identify lymphocytes based upon size and granularity (**A**), CD3 was used to obtain the T cell population (**B**), percentage of CD3 cells that were CD4+ was identified in (**C**). Alternatively, CD3 x CD4 gating was performed on the FSC, SSC subset to determine the percentage of all lymphocytes that were CD3+CD4+ (**D**).

**
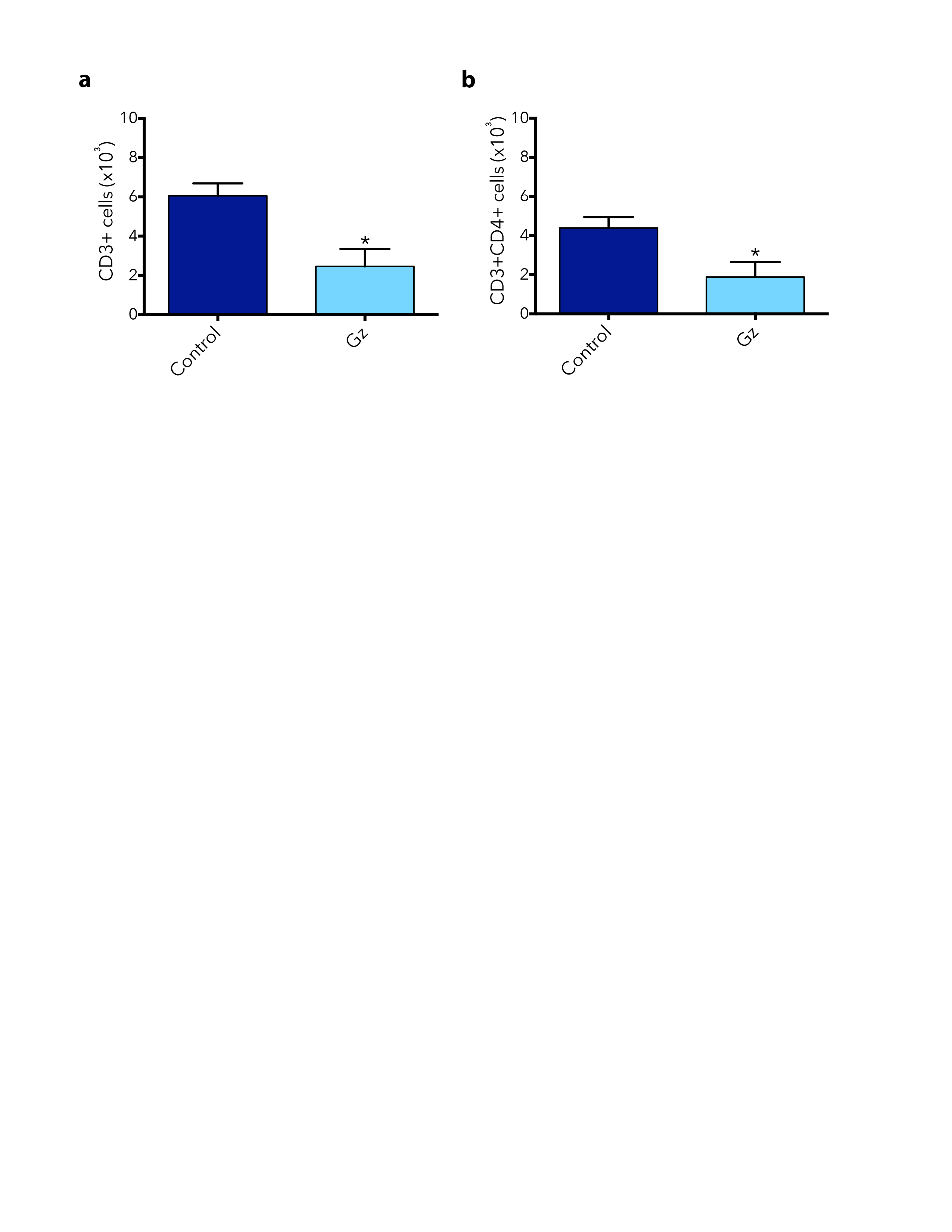
**

**Figure S5. Absolute numbers of T cell numbers in the spinal cord of untreated versus Gz-treated EAE mice.** Spinal cords isolated from control or Gz-treated EAE animals were isolated at Day 21 post-MOG immunization. Tissue was digested, myelin removed by Percoll centrifugation, and the single cell suspension was stained with CD3 and CD4. Total number of CD3+ cells are shown in (**A**) and total number of CD3+CD4+ cells in (**B**). Data are mean ± SEM. n = 4-6. *p<0.05.

**Figure S6. Guanabenz does not alter iNOS expression in primary microglia.** Primary microglia were treated with saline, IFNγ, 10 μM Gz, or IFNγ and 10 μM Gz concomitantly. Cells were fixed on coverslips and stained with iNOS (red) and Iba1 (green). DAPI (blue) marks cell nuclei (**A**). The percent of cells expressing iNOS in each field was quantified manually (**B**). Data preented are mean ± SEM. n = 3 technical replicates in 2 independent experiments.

|  | **Saline** | **IFNγ** | **Gz** | **IFNγ+Gz** |
| --- | --- | --- | --- | --- |
| **Saline** | 1 | 0.00979 | 1.64 x 10^-8^ | 0.000597 |
| **IFNγ** |  | 1 | 5.55 x 10^-16^ | 1.13 x 10^-8^ |
| **Gz** |  |  | 1 | 0.0564 |
| **IFNγ+Gz** |  |  |  | 1 |

**Supplementary Table S1. Phagocytosis assay p-values derived from K-S test.** Integrated density of the red pixels within each cell were analyzed using a K-S test via https://github.com/mcap91/Monocyte-Phagocytosis-Assay.
